# New Postal Law

**Published:** 1875-01

**Authors:** 


### The Bistoury.

ELMIRA, N. Y., JAN., 1875.

The BISTOURY is published Quarterly, upon the 1st of April, July, October and January, at Fifty Cents a year, in advance.

### NEW POSTAL LAW.

After the first of January, the new postal law, requiring publishers to pre-pay postage upon newspapers and magazines, goes into effect. Accordingly, commencing with the next number of the BISTOURY, we will be required to pre-pay the postage upon every number of our magazine. In order to meet this requirement, our subscribers will please remember to enclose an extra *five cents* for the BISTOURY for the coming year. Canadian subscribers will be charged *ten cents* for postage, making the subscription price 55 cents for all points in the United States, and 60 cents in Canada. The price of our journal is so low that we cannot undertake to meet the extra expense of postage. Five cents, to an individual, is a small matter, but when several thousand five cents are required of the publisher, it amounts to a considerable sum of money.

Enclosed will be found the usual yearly envelope and blank, in which the subscription can be sent. All subscriptions received, will be promptly acknowledged, in the usual place, in the April number. Do not ask us to mail you a receipt, we cannot do it, as it occupies too much time, more than our mailing clerk has to devote to such a purpose.

Extending to all our readers the usual compliments of the season, we engage to send them the BISTOURY, for the eleventh year, as brim-full of useful information as in years gone by.

